# Beyond sex allocation: the role of mating systems in sexual selection in parasitoid wasps

**DOI:** 10.1111/brv.12126

**Published:** 2014-07-01

**Authors:** Rebecca A Boulton, Laura A Collins, David M Shuker

**Affiliations:** 1 Centre for Biological Diversity, School of Biology, University of St AndrewsDyers Brae, Greenside place, Fife KY16 9TH, U.K.

**Keywords:** parasitoid, sexual selection, mating system, sperm depletion, sex allocation

## Abstract

Despite the diverse array of mating systems and life histories which characterise the parasitic Hymenoptera, sexual selection and sexual conflict in this taxon have been somewhat overlooked. For instance, parasitoid mating systems have typically been studied in terms of how mating structure affects sex allocation. In the past decade, however, some studies have sought to address sexual selection in the parasitoid wasps more explicitly and found that, despite the lack of obvious secondary sexual traits, sexual selection has the potential to shape a range of aspects of parasitoid reproductive behaviour and ecology. Moreover, various characteristics fundamental to the parasitoid way of life may provide innovative new ways to investigate different processes of sexual selection. The overall aim of this review therefore is to re-examine parasitoid biology with sexual selection in mind, for both parasitoid biologists and also researchers interested in sexual selection and the evolution of mating systems more generally. We will consider aspects of particular relevance that have already been well studied including local mating structure, sex allocation and sperm depletion. We go on to review what we already know about sexual selection in the parasitoid wasps and highlight areas which may prove fruitful for further investigation. In particular, sperm depletion and the costs of inbreeding under chromosomal sex determination provide novel opportunities for testing the role of direct and indirect benefits for the evolution of mate choice.

## I. Introduction

Since the 1970s, the study of sexual selection has come to dominate much of behavioural ecology (Andersson, 1994). Building on key advances by Parker (1970), Trivers (1972), Emlen & Oring (1977), Lande (1981) and Kirkpatrick (1982), behavioural ecologists have made tremendous progress in understanding the causes and consequences of sexual selection across a whole range of organisms (Andersson, 1994; Shuker, 2010; Davies, Krebs & West, 2012). Many species of insects have played their part in the resurgence of interest in sexual selection and the evolution of mating systems (Blum & Blum, 1979; Thornhill & Alcock, 1983; Choe & Crespi, 1997; Shuker & Simmons, 2014), particularly in terms of post-copulatory sexual selection (Parker, 1970; Eberhard, 1996; Simmons, 2001) and the links between sexual selection and sexual conflict over mating (Rowe *et al.*, 1994; Arnqvist & Rowe, 2005). Some insect orders have clearly played a greater role than others though, in particular Orthoptera (especially various bushcrickets and gryllid crickets) and Diptera (especially drosophilids and dung-flies). By comparison, rather less has been done in terms of the study of sexual selection and sexual conflict in the parasitic Hymenoptera, even though they have a diverse array of mating systems (Godfray & Cook, 1997). Instead, parasitoid mating systems have typically been studied by evolutionary ecologists in the context of how population structure influences sex allocation strategies, producing a prodigious body of work (Hamilton, 1967, 1979; Charnov, 1982; King, 1996; Godfray, 1994; most recently summarised by West, 2009). Furthermore, as crucial biological control agents, parasitoid wasps have been the focus of much applied research, with work on host choice, mating systems and sex allocation undertaken in order to facilitate the improved attack and control of pest populations (e.g. Wajnberg, Bernstein & van Alphen, 2008, and papers therein).

**Figure 1:**
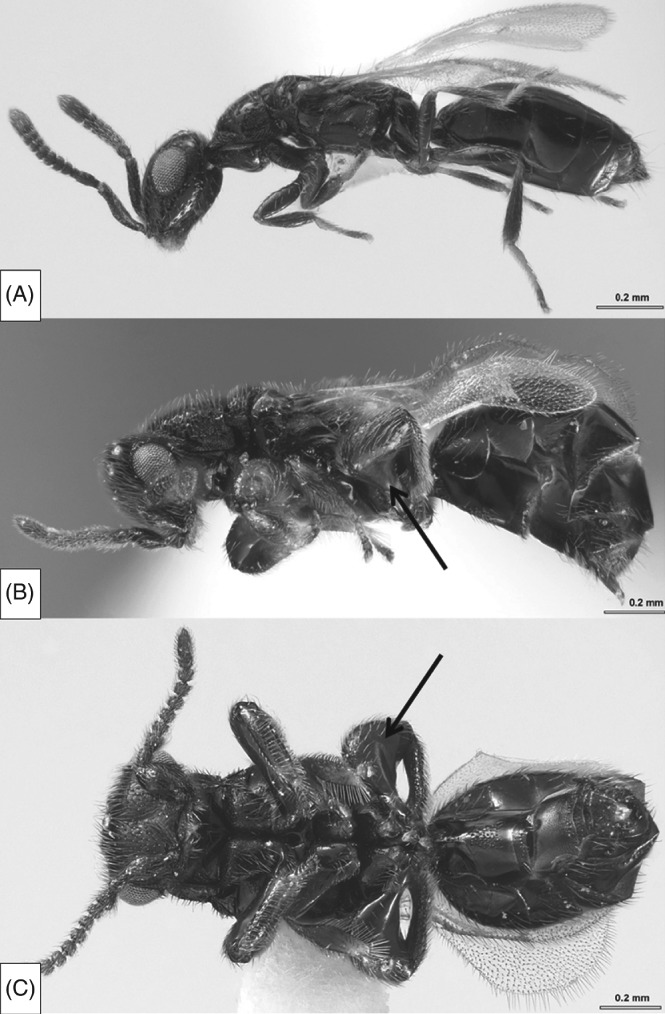
*Spalangia dozieri,* habitus: (A) ♀, lateral view; (B) ♂, lateral view; (C) ♂, ventral view (from Gibson & Reigada, 2009). Arrows highlight sexually dimorphic ‘grasping’ legs of males.

Recently however there have been a number of research groups re-visiting aspects of the mating biology of parasitoid wasps, revealing that there may yet be much scope for sexual selection to act in this taxon. Indeed, some may provide useful model species for the study of aspects of sexual selection and sexual conflict over mating. These include the benefits of mate choice, the interaction between pre- and post-copulatory sexual selection, and the evolutionary causes and consequences of female multiple mating (polyandry). Table 1 lists empirical studies which have sought explicitly to test hypotheses relating to sexual selection in both males and females across the parasitic Hymenoptera. This table does not represent an exhaustive list of everything known about sexual selection in the parasitoids; many more studies have been conducted (some of which this review will cover), but not under the framework of sexual selection. Out of the 39 studies included in the table, only four were conducted prior to 2000, and the majority after 2007. We hope that this reflects emerging interest in parasitoid sexual selection and that this review may encourage those who share this interest to pursue it further. Whilst it may be true that parasitoids appear to lack some of the extravagant ornaments or displays of other insects [such as the wonderful dolichopodid fly *Poecilobothrus nobilitatus* (Dolichopidae): Zimmer, Diestelhorst & Lunau, 2003] there are species with elaborate sexual dimorphism [e.g. *Spalangia dozieri* Burks (Pteromalidae); Gibson & Reigada, 2009; Fig. 1] that may prove to be under sexual selection, and even commonly studied species like *Nasonia vitripennis* are clearly dimorphic in terms of their cuticular iridescence, with males much more iridescent than females. As such, a greater appreciation of the mating behaviour and the possibilities for sexual selection may yet reveal examples of a parasitoid ‘peacock's tail’ (Godfray, 1994).

**Table 1:** Studies of sexual selection in parasitoid wasps

Species	Family	Selective mechanism	Method	Fitness measure	Trait	Results	Gregarious/solitary	Spermatogeny	Ovigeny	Reference
*Aphelinus asychis*	Aphelinidae	M-CH	LE	M-Response	F-Mated status	M responded to virgin not mated F pheromones	S	?	?	Fauvergue *et al.* (1995)
					F-Pheromones					
*Cephalonomia tarsalis*	Bethylidae	M-CO	LO	M-Cop success	M-Age	Older males have reduced cop success, no effect of body size on cop success. Smaller males have lower cop duration but no effect on SR	S	P	—	Cheng *et al.* (2003)
					M-Body size					
*Cephalonomia tarsalis*	Bethylidae	F-CH/M-CH	LO	M-Cop success	M-Mated status	No CH by either sex based on mated status (CH & NCH tests)	S	P	?	Cheng *et al.* (2004)
				F-SR	F-Mated status	F remating did not affect SR				
*Cephalonomia hyalinipennis*	Bethylidae	F-Fit cost F-RM	LO	F-Fecundity	M-Mated status	Males become sperm depleted	G	P	S	Pérez-Lachaud (2010)
						Mating with a sperm-depleted M does not influence F fecundity but reduces daughter production (SR)				
				F-SR	F-Remating	F remating allowed daughter production to resume				
*Diaeretiella rapae*	Braconidae	F-RM	LO	F-fecundity	M-mated status	M remating occurs and results in sperm depletion which reduces F fecundity	S	P	?	Kant *et al.* (2012)
		M-RM		F-SR	F-Mated status	No F remating				
*Diachasmimorpha longicaudata*	Braconidae	F-RM	LO	M-Rep success (eye colour)	F-Remating	No sperm precedence unless second mating occurs 24 h after first (second male precedence)	S	?	?	Martínez-Martínez, Leyva-Vázquez & Mojica (1993)
		M-Sp comp								
*Glyptapanteles flavicoxis*	Braconidae	F-CH	LO	M-Response	M-Wing-fanning	F attracted to M wing-fanning	G	?	?	Danci *et al.* (2010)
		M-CH		F-Response	F-Flight sounds	M attracted to F flight sounds particularly when presented with F pheromone				
					F-Pheromones					
*Cardiochilesnigriceps*	Braconidae	M-Cop success	LO	M-Cop success	M-Age	M advantage to early emergence	S	?	?	Hirose & Vinson (1988)
		F-CH								
*Cotesia marginiventris*	Braconidae	F-CH	LO	Cop success	M-Body size	F preference for large M	S	?	?	Joyce *et al.* (2009)
		M-CH			F-Body size	No M preference for F size				
*Cotesia flavipes*	Braconidae	F-CH	LO	Cop success	M-Body size	No F size preference	G	?	?	Joyce *et al.* (2009)
		M-CH			F-Body size	M preference for small F				
*Aphidius ervi*	Braconidae	F-CH	LE	M-Cop success	M-Mated status	M synspermatogenic	Q-G	S	?	He & Wang (2008)
				F-SR	M-Age	Once mated M have higher cop success than virgin M				
					F-Age	Old M & F produce fewer daughters				
						M & F preference for younger mates				
*Venturia canescens*	Ichneumonidae	F-CH	LO	F-Response	M-Relatedness	Kin discrimination by F only in NCH tests (not CH)	S	?	S	Metzger *et al.* (2010)
						When exposed to sib-male extract F less likely to mate with unrelated male				
*Spalangia cameroni*	Pteromalidae	F-CH	LO	M-Cop success	M-Mated status	M still attempt copulation when sperm depleted	S	P	?	King (2000)
						Virgin F do not discriminate sperm-depleted and non-sperm-depleted M				
*Spalangia endius*	Pteromalidae	M-CH	LE/LO	F-Cop success	F-Mated status	M preference for virgin F (CH and NCH tests)	S	P	?	King *et al.* (2005)
*Spalangia endius*	Pteromalidae	M-CH	LO	M-Response	F-Mated status	M aversion to living mated but not virgin F	S	P	?	King & Dickenson (2008)
						No preference/aversion to dead virgin or mated F				
*Spalangia endius*	Pteromalidae	F-Fit cost	LE	F-Fecundity	M-Mated status	Sperm depletion costly to females	S	P	?	King & Bressac (2010)
		F-RM		F-SR	F-Remating	No benefit to F remating in terms of fecundity, SR or longevity				
				F-Longevity						
*Spalangia endius*	Pteromalidae	F-Fit cost	LE/LO	F-SR	M-Mated status	Daughter production reduced when F mated with M who had mated five times previously	S	P	?	King & Fischer (2010)
		F-CH		M-Cop success		Virgin M have higher cop success due to motivation, not F-CH				
*Spalangia endius*	Pteromalidae	M-CH	LE	M-Response	F-Mated status	M temporary aversion to copulating after mating or encountering a mated F	S	P	?	Fischer & King (2012)
*Nasonia spp*	Pteromalidae	M-CO	LO	F-RM	Species	F of *N. vitripennis* most likely to remate	G	P	S	Leonard & Boake (2008)
		F-RM		M-Courtship		Longer pre-cop courtship associated with reduced F remating in *N. vitripennis* and *N. longicornis*				
						Longer post-cop courtship associated with reduced F remating in *N. giraulti*				
*Nasonia spp*	Pteromalidae	F-RM	LO	F-Fecundity	Species	Mating first with a heterospecific iM increased probability of F remating with a conspecific	G	P	S	Geuverink *et al.* (2009)
				F-SR		No effect of mating on F longevity				
				F-Longevity						
				F-RM						
*Nasonia vitripennis*	Pteromalidae	F-RM	LO	F-RM	Strain	F from strains maintained in the laboratory for longer had increased F remating	G	P	S	Burton-Chellew *et al.* (*a*)^2007^
						Experimental crosses indicate this results from F not M behavioural changes				
*Nasonia vitripennis*	Pteromalidae	M-CO	LO	M-Cop success	M-Body size	M body size had no effect on cop success even in the presence of a competitor	G	P	S	Burton-Chellew *et al.* (*b*)^2007^
				M-Longevity	(M–CO)	Mating reduced M longevity				
						M body size did not affect M longevity				
*Nasonia Vitropennis*	Pteromalidae	F-CH	LE	F-Response	F-Mated status	Only virgin F show a preference for M pheromone	G	P	S	Ruther *et al.* (2007)
					M-Pheromones					
*Nasonia vitripennis*	Pteromalidae	M-CO	LO	M-Cop success	M-Age	Large M emerge earlier	G	P	S	Moynihan & Shuker (2011)
		F-CH			M-Body size	Early-emergence mating advantage outweighs size advantage				
*Nasonia vitripennis*	Pteromalidae	M-CO	LO/LE	M-Pheromones	M-Body size	Large M produce more pheromone and are more attractive to F	G	P	S	Blaul & Ruther (2012)
		F-CH		M-Cop success		Small M had increased cop success in direct competition				
				F-Response						
*Nasonia vitripennis*	Pteromalidae	F-CH	LE	M-Pheromones	M-Mated status	Sperm-depleted M produce less pheromone	G	P	S	Ruther *et al.* (2009)
				F-Response		F preference for pheromones of non-sperm-depleted M				
*Dinarmus basalis*	Pteromalidae	F-Fit cost	LO	F-Sp store	F-Remating	Three-times-mated F stored more sperm and produced more daughters than once-mated F	S	P	S	Chevrier & Bressac (2002)
				F-SR						
		F-RM		F-Fecundity		No fecundity differences in once- and three-times-mated F				
*Dinarmus basalis*	Pteromalidae	M-CO	LE	M-Body size	Host size	M reared on small hosts were smaller with less sperm	S	P	S	Lacoume, Bressac & Chevrier (2006)
						Small M disadvantage in competitive situations				
		M-Fit cost		M-Cop success		Non-competitive situations no small M disadvantage				
		F-CH		M-Sp store		No evidence of F CH				
*Anisopteromalus calandrae*	Pteromalidae	F-RM	LO	F-Sp store	F-Remating	No effect of double mating on F Sp store, F fecundity or F SR	S	?	?	Khanh, Bressac & Chevrier (2005)
		M-Sp comp		F-Fecundity	M-Strain	No F preference for red-eye or wild-type males				
				F-SR		No sperm precedence based on mating order				
				M-SR		Red-eye M produce more sperm than wild-type which is reflected in daughter production				
*Pteromalus venustus*	Pteromalidae	F-Fit cost	LO	F-SR	M-Mated status	No relationship between M mating status and SR	G	?	?	Tepedino (1993)
						M insemination capacity is double the number of F they share a host with				
*Lariophagus distinguendus*	Pteromalidae	M-CH	LE	F-Fecundity	M-Mated status	Reduced daughter production when mating with a sperm-depleted M	G	P	?	Steiner *et al.* (2008)
		F-CH		F-SR		M mated status did not influence the probability that a F would remate				
				F-RM						
*Lariophagus distinguendus*	Pteromalidae	M-CO	LO	M-Response	Pupae sex	Developing M produce the same pheromone as developing F	G	P	?	Steiner *et al.* (2005)
				M & F-Pheromones		Emerged M exhibit courtship towards pupae of either sex				
						Late-emerging M could benefit by distracting early emerging M				
*Lariophagus distinguendus*	Pteromalidae	F-CH	LO	M-Cop success	M-Wing-fanning	Closer proximity to F and higher frequency wing-fanning more likely to result in successful copulation	G	P	?	Benelli *et al.* (2013)
*Urolepis rufipes*	Pteromalidae	M-CO	LO	F-RM	M-Courtship	Post-cop attendance by M did not reduce the probability of F remating but did increase daughter production	S	?	?	King & Kuban (2012)
				F-SR						
*Trichogramma euproctidis* (previously *T. evanescens)*	Trichogrammatidae	M-Fit benefit	LO	F-Sp store	M-Mated status	F which remate after mating with a sexually dimorphic M store less	Q-G	S	P	Damiens & Boivin (2005)
					F-Remating	Sperm-depleted males which continue to mate could increase their relative fitness				
*Trichogramma euproctidis* (previously *T. evanescens)*	Trichogrammatidae	F-Fit benefit	LO	F-Fecundity	F-Remating	No effect of polyandry on F fecundity or F SR	S	S	P	Jacob & Boivin (2005)
				F-SR		Multiple mating increased F longevity but only in the presence of hosts				
				F-Longevity						
*Trichogramma euproctidis* (previously *T. turkestanica)*	Trichogrammatidae	M-CH	LE	M-Response	F-Mated status	M preference for virgin F	Q-G	S	?	Martel *et al.* (*a*)^2008^
					M-Age	No effect of age on M choosiness				
					M-Fed or unfed	Unfed M choosier than fed M				
*Trichogramma euproctidis* (previously *T. turkestanica)*	Trichogrammatidae	M-CO	LO	F-SR	M-CO	M transfer less sperm when increased competition reduces sperm wastage due to first male precedence and prospermatogeny	Q-G	S	?	Martel, Damiens & Boivin (*b*)^2008^
*Uscana semifumipennis*	Trichogrammatidae	F-Fit cost	LO	F-Fecundity	M-Body size	F mated to larger M produced more daughters	S	P	?	Henter (2004)
				F-SR						

Selective mechanism: F, female; M, male; CH, choice; CO, intra-sexual competition; Cop success, copulation success; Fit benefit, fitness benefits; Fit cost, fitness costs; RM, remating; Sp comp, sperm competition.

Method: LE, laboratory experiments; LO, laboratory observations.

Fitness measure: Cop success, copulation success; Rep success, reproductive success; RM, remating; Response, sexual response; Sp store, sperm storage; SR, sex ratio (daughter production).

Results: NCH, no-choice test; CH, choice test.

Trait: Body size, a measure of body size; Mated status, # previous mates; Strain, laboratory strain.

Gregarious/solitary: G, gregarious; Q-G, quasi-gregarious; S, solitary.

Spermatogeny: P, prospermatogenic; S, synspermatogenic; ?, unknown.

Ovigeny: P, proovigenic; S, synovigenic.

In this review we aim first to outline some key aspects of parasitoid biology that are crucial to understanding their mating systems and the possibilities for sexual selection, particularly in terms of the costs and benefits of female mate choice and multiple mating. Second, we will describe some of the main mating systems observed across parasitoids. Third, we will briefly outline the role parasitoid mating systems play in terms of patterns of sex allocation, due to the importance of (*i*) the operational sex ratio and (*ii*) interactions among kin for sexual selection and sexual conflict over mating. Fourth, we will review what we know about sexual selection in parasitoid wasps, both before and after copulation, and highlight promising avenues for future research. By so doing, we hope to highlight opportunities for behavioural ecologists that neglected organisms such as parasitoid wasps may offer for clarifying existing problems in sexual selection.

## II. Basic Biology of Parasitoid Wasps

### (1) The parasitoid lifestyle

Parasitoids are animals that parasitise other organisms, but in doing so also destroy their hosts (typically by eating many or all of the body tissues). As such, parasitoids share aspects of both the parasite and predator lifestyle; like predators and unlike parasites they kill their hosts, unlike predators they have only one victim during their life (Godfray, 1994; Lafferty & Kuris, 2002). The vast majority of parasitoids are solitary (non-social) wasps (Hymenoptera), although other insects including dipteran flies (e.g. the Tachinidae), Strepsiptera (the so-called twisted-wing parasites), beetles (e.g. some Staphylinidae), Lepidoptera and the Neuroptera (including the Mantispidae and Symphrasinae) and the Trichoptera also have a parasitoid lifestyle (Mills, 2009). We will focus mainly on parasitoid wasps with invertebrate hosts. Parasitoids may be either ectoparasitic (attaching to the outside of the host) or endoparasitic (consuming the host from within). All invertebrate life stages are attacked (egg, larva or nymph, pupa and adult) and parasitoids can be classified as koinobionts (their hosts continue to develop and grow to some extent, such as larvae) or idiobionts (where hosts will not grow further, e.g. pupae). Parasitoids may attack a host by ovipositing on or in the host, or depositing a larva on or near a host. Parasitoids may be solitary (one parasitoid per host), quasi-gregarious (one parasitoid per host, but hosts are spatially clumped, such as a clutch of eggs on a leaf), or gregarious (multiple parasitoids per host; they may be from the same mother or from different mothers, a situation termed superparasitism if the mothers are from the same species, or multiparasitism if they are from different species: Waage, 1986; Godfray, 1994). Given this background, clearly the biology and distribution of their hosts will play a major role in defining the distribution of the parasitoids themselves, and thus their mating systems (Emlen & Oring, 1977).

### (2) The consequences of haplodiploidy

In addition to their parasitic lifestyle, parasitoid wasps are also haplodiploid: males develop from unfertilised eggs and are haploid, whilst females develop from fertilised eggs and are diploid (Cook, 1993; Heimpel & de Boer, 2008). There are exceptions, such as parthenogenetic, female-only strains and species, but we will focus here on arrhenotokous haplodiploids. This genetic system means that males have no father, with a maternal grandfather their closest male progenitor. Daughters are therefore the only route for genetic transmission for male alleles, creating the opportunity for sexual conflict over genetic transmission (Shuker, Moynihan & Ross, 2009). Whilst the origins of haplodiploidy remain unclear [see Normark (2004, 2006) and Ross, Pen & Shuker (2010) for recent discussions], some of the consequences are better known. For instance, haplodiploidy is proposed to reduce the genetic load of deleterious alleles, and thus reduce inbreeding depression. The reasoning is that males, being haploid, express any deleterious recessives, exposing them to selection, and so these alleles are more effectively purged than in diplo-diploids. Although the reasoning is sound, inbreeding depression is not entirely absent in parasitoids (or indeed other Hymenoptera; Henter, 2003), but there are certainly many examples of extreme inbreeding without deleterious effects in parasitic wasps (e.g. most gregarious parasitoids: Godfray, 1994). Haplodiploidy, in combination with inbreeding, also changes the patterns of relatedness between family members, which should (at least theoretically) influence patterns of sex allocation and also influence family conflicts. The asymmetric nature of the haplodiploid genetic system also changes how indirect selection can work, in a manner directly analogous to the sex chromosomes. In the most obvious example, sons do not inherit their fathers' genes, and so mechanisms of indirect sexual selection (be they Fisherian or ‘good genes’ processes) cannot work in such a straightforward way.

Reeve & Pfennig (2003) modelled sexual selection on extravagant male traits across different genetic systems (diploidy with male *versus* female heterogamety and haplodiploidy). Their model indicated that male secondary sexual characters should be less well developed in haplodiploids compared to diploids and they presented empirical data in support of this. A key aspect of their analysis considered the extent to which haplodiploidy increased the exposure of rare alleles coding for male traits and/or female preferences to loss by drift. Their findings seem intuitive when we consider how mechanisms of indirect sexual selection and genetic systems might interact. For instance, in diploids, during Fisherian runaway sexual selection (Fisher, 1930; Lande, 1981; Kirkpatrick, 1982) a male secondary sexual ornament will increase in frequency due to a female preference for that trait. Offspring of males bearing the trait and females exhibiting the preference will inherit both, resulting in a genetic covariance between the preference and the ornament and thus a positive feedback between the two, leading to an increase in frequency of both the trait and preference. In haplodiploids, males have no fathers and so a Fisherian process cannot occur in the same way. Of course, male genes are not ‘lost’. For instance, we do know that while in the parasitoid wasp *Nasonia* (Pteromalidae), male hybrids between *N*. *vitripennis* (Walker) and *N*. *longicornis* (Darling) exhibit male courtship displays that are intermediate between the parental species, there is nonetheless a ‘grandfather effect’; males courtship displays most resemble that of the grand-paternal species (Beukeboom & van den Assem, 2001, 2002). However, the build-up of the necessary genetic covariance between preference and trait is constrained under haplodiploidy and further theoretical work would be informative to help clarify the strength of indirect selection in this case.

The ‘good genes’ model of sexual selection may be more relevant to haplodiploids however. If male secondary sexual traits indicate quality or viability which is heritable, a female mating with a high-quality male may produce female offspring of greater viability (i.e. survivorship and fecundity). Even though in haplodiploids only daughters will inherit these ‘good genes’, if the female preference is heritable and the ‘good genes’ increase female offspring performance, we might expect the preference to increase in frequency.

However, perhaps the most important aspect of haplodiploidy is in terms of sex determination itself. Beyond the differences in ploidy, the way sex is actually determined varies throughout the parasitoid Hymenoptera. Single-locus complementary sex determination (sl-CSD) occurs when a single genetic locus determines the sex of the individual that it is found in. If an individual has only a single allele it will develop into a male (this male can be haploid, i.e. hemizygous, or diploid and homozygous at this locus). A heterozygote will develop as a female (Whiting, 1943; Cook, 1993; Zhishan *et al.*, 2003; Heimpel & de Boer, 2008). Whiting (1943) first found evidence of this system in the braconid parasitoid *Habrobracon* (previously *Bracon*) *hebetor* (Say). Multi-locus CSD occurs when there are two or more independent sex loci that dictate whether an individual will develop as male or female; this has been found in other braconid species such as *Cotesia vestalis* Haliday (Braconidae) (Holloway *et al.*, 2000; de Boer *et al.*, 2007). Evidence of CSD (single and multi-locus) in the parasitoids has, thus far, been found only in members of the superfamily Ichneumonoidea. Crucially, diploid males are in many cases either inviable or sterile (Cook, 1993) and so are costly for a mother to produce or for a female to mate with (Heimpel & de Boer, 2008). Since inbreeding will lead to the increased homozygosity of diploid individuals, and so greater production of diploid males, it may be extremely costly in species with CSD. We will see below the consequences this might have on reproductive behaviour. However, as mentioned above, inbreeding can often be extreme, without diploid male production being the result (e.g. in many chalcidoid and bethylid wasps no diploid males are seen: Zhishan *et al.*, 2003). Across these species, one or more different sex-determination mechanisms may be operating such as genomic imprinting seen in speices such as *Nasonia vitripennis* where haploid eggs contain only a maternally imprinted (and silenced) copy of the *transfomer* gene and develop into males (diploid eggs contain an additional non-imprinted paternal copy which is activated on fertilisation and leads to the development of diploid females; Verhulst, Beukeboom & van de Zande, 2010).

## III. Mating Systems of Parasitoid Wasps

Mating systems are shaped largely by the spatio-temporal predictability of reproductively capable females and the ability with which one sex (often males) can monopolise the other (typically females) either directly (for instance in terms of forming harems) or indirectly, by monopolising access to key resources (including sperm: Emlen & Oring, 1977). Mating systems can also be conceptualised in terms of the opportunity for selection (e.g. Shuster & Wade, 2003), although similar classifications of mating systems tend to result. In parasitoids, mating systems may be difficult to categorise and often display varying levels of overlap (Godfray, 1994). The classification of parasitoid mating systems has typically been fairly simplistic compared to that in other taxa, probably because the predominant focus is sex allocation (Hardy, Ode & Siva-Jothy, 2005). For parasitoid wasps, the distribution of host species is the key ecological determinant underlying the availability and defensibility of mates (Emlen & Oring, 1977). Unmated individuals will emerge from hosts and females will seek unparasitised hosts for oviposition, providing potential mating opportunities either at the emergence site, or over broader spatial scales depending on species and environment-specific characteristics.

As outlined above (Section II.1^2006^), parasitoids vary in their tendency to aggregate. The mating systems of gregarious and quasi-gregarious parasitoids often involve mating at the emergence site. Males typically emerge first and wait to mate with emerging females (protandry; Godfray, 1994; Godfray & Cook, 1997). This system often results in sibmating (unless there is selection against inbreeding; Section II.2^1994^) and in extreme cases mating occurs within the host prior to emergence (Godfray & Cook, 1997). For instance, the level of so-called within-host mating (WHM) varies across species of *Nasonia*, occuring most frequently in *Nasonia giraulti* Darling (Pteromalidae) and least in *Nasonia vitripennis* (Drapeau & Werren, 1999). There may also be fatal male–male competition within hosts prior to emergence (Hamilton, 1979), as in the case with *Melittobia* wasps (Abe *et al.*, 2003). When WHM does not occur, protandrous males may go as far as assisting female emergence to secure matings, for instance in *Trichogramma papilionis* (Suzuki & Hiehata, 1985). In some species males appear to wait on hosts from which females are about to emerge (e.g. *N. vitripennis*, although the cues used by males are currently unknown: Shuker *et al.*, 2005). *N. vitripennis* males also defend temporary territories around emergence holes, although these territories tend to break down into scramble competition under high population density (van den Assem, Jachmann & Simbolotti, 1980).

Generally it is expected that when males and females do not emerge at the same location (as in many solitary species), or if sibmating is detrimental, early-emerging males may be attracted to sites where females are soon to emerge. In such cases long-range female pheromonal cues and chemicals produced by hosts (and associated symbionts) may be particularly important as male attractants. However, there are so far relatively few examples of long-range pheromones in parasitoid wasps. One possible example comes from Davies & Madden (1985) who found evidence of volatile female mandibular pheromones which are thought to be involved in male aggregation in *Megarhyssa nortoni* Cresson (Ichneumonidae) and *Rhyssa persuasoria* L. (Ichneumonidae) and also in *Megarhyssa* sp. (Matthews, Matthews & Crankshaw, 1979). On the other hand, the absence of volatile long-range pheromones in the gregarious scale insect parasitoid *Aphytis melinus* De Bach (Aphelinidae), coupled with the deposition of a sex pheromone trail, suggests that males mate primarily in the aggregation from which they emerged (Bernal & Luck, 2007). Fauvergue, Hopper & Antolin (1995) also found evidence of sex pheromone trails in a solitary species, the aphid parasitoid *Aphelinus asychis* Walker (Aphelinidae). The reason for the absence of long-range volatile pheromones in *A. asychis* was initally unclear as this solitary parasitoid typically occurs at low densities. However the model proposed by Fauvergue *et al.* (1995) demonstrated that this system should result in most females being mated at densities typically observed in the field (2.2 males per m^2^ resulted in 100% of 2-day-old females being mated). At lower densities (of around 0.3 males per m^2^, seen early in the season) 50% of females will remain unmated after 2 days, but after 5 days a virgin female would have a 95% probability of being mated. Godfray (1994) suggested that the high metabolic costs of pheromone production, combined with the haplodiploid genetic system which means that females do not need to be inseminated to begin reproduction (due to haplodiploidy virginity being costly, but not fatal for fitness), may have contributed to the rarity of long-distance pheromones in parasitoids.

One further possibility is that males may be able to use kairomones produced by hosts to find locations where mates are likely to be. Similarly, the ability to reproduce as virgins means that females can perhaps benefit from seeking hosts before they have mated. Males and females may both, therefore, seek oviposition sites in order to come together for mating. The majority of studies on the use of host kairomones thus far though have focused on females finding oviposition sites. For instance, *Habrobracon hebetor* females (but not males) are attracted to the sex pheromones produced by males of its host, the greater waxmoth [*Galleria mellonella* L. (Pyralidae), Dweck *et al.*, 2010]. One study which did investigate the use of kairomones by males found that in *Spalangia cameroni* Perkins (Pteromalidae), a solitary parasitoid of house fly pupae, males are attracted to odours produced by suitable hosts and host environment odours (chicken manure; Myint & Walter, 1990). Godfray (1994) and Godfray & Cook (1997) suggested that mating will be more likely to occur at oviposition sites when females are not receptive on emergence and also mate multiply, with the last male to mate having the greatest fertilisation success. It is as yet unclear whether cues from hosts are used for mate finding in other species, whether males and females are capable of utilising the same cues, and how this ability relates to the mating system under study as well as other life-history variables or patterns of sperm competition.

Alternatively, males may aggregate around other resources which females require in order to reproduce. Although hosts often represent both feeding and oviposition sites, many parasitoid wasp species do utilise other food sources, such as flowers that provide nectar. As a consequence, it has been proposed that some species will mate locally at feeding sites when these are well defined and females feed predictably. Females of such species may be synovigenic; they are unable to mature their eggs until they feed, and so may not be receptive upon emergence (Godfray, 1994; Godfray & Cook, 1997). The extent of this mating system is currently unclear; a review by Jervis *et al.* (1993) found that in 18.5% of the 250 species recorded, both males and females were found at flowers, but in none of these instances was copulation recorded.

Males may also aggregate at sites which contain no obvious resources: their sperm is the resource and females visit them with the sole intention of obtaining copulations (Emlen & Oring, 1977). A common form of temporary mating aggregation in insects is termed ‘hill-topping’, when males and females aggregate at landmarks or at the top of geographic features (such as hills, or even tall man-made structures) that are visible from relatively long distances (Parker, 1978; Thornhill & Alcock, 1983). For instance, males of *Hemipepsis ustulata* Dahlbom (Pompilidae), the tarantula hawk wasp, defend trees (which do not contain resources that females can utilise) occurring on mountainous ridges. This mating system most likely arose when females are never spatially aggregated, and so other strategies are unprofitable for males (Thornhill & Alcock, 1983). The higher and more prominent trees are more desirable, and the largest and most aggressive males acquire and defend them (Alcock & Kemp, 2006). This system does suggest that female choice plays a part in the mating system of *H. ustulata* and it might be beneficial for females to mate with the ‘top’ males which possess superior endurance and flight ability, if these traits have a heritable component (Thornhill & Alcock, 1983).

Alternatively, these arbitrary mating aggregations may be more structured, with males defending small, resource-free display territories, also known as leks (Höglund & Alatalo, 1995). When the lek is an aerial aggregation, it is typically called a swarm (Godfray, 1994; Sivinski & Petersson, 1997). Generally, mating systems of this kind are thought to have evolved when there are ecologically few opportunities to monopolise resources or females. In parasitoids, resource-free mating systems may arise for instance when inbreeding is deleterious among gregarious or semi-gregarious species, or when the species is solitary and highly dispersed thanks to the distribution of their hosts, such that an aggregation will be easier to find than an individual (Godfray & Cook, 1997).

Swarms and leks have been observed in various braconids and chalcidoids. For example, *Habrobracon hebetor,* a gregarious parasitoid of pyralid moth caterpillars which live on stored products such as grain, forms terrestrial aggregations composed of 80% males on the peaks of infested corn piles (Antolin & Strand, 1992). The aggregations contain no resources and therefore fit the definition of a lek, however, no aggression was observed between males nor is it certain that these sites actually function as display arenas. As such, this mating system was categorised as male scramble competition polygyny, although as yet the potential for female choice has not been investigated. Godfray & Cook (1997) highlighted the need for more detailed field observations of such systems, particularly considering the potential for male–male competition and female choice, a recommendation that still needs to be followed. Insect leks have not, in general, been studied as much as they deserve (Niyazi, Shuker & Wood, 2008), and many tests of lek formation theory remain to be carried out.

## IV. Sex Allocation in Parasitoid Wasps

Sex allocation is the allocation of resources to male *versus* female offspring. The literature on sex allocation in parasitoid wasps is extensive (e.g. West, 2009), a mark of the enormous predictive success of the theory base initiated by Hamilton (1967, 1979). Much of the work on sex allocation in parasitoids focuses on offspring sex ratios, i.e. the numbers of male and female offspring mothers produce. Throughout we will consider sex ratio as the proportion of offspring that are males. Here we will only provide a very brief summary of the theory. Importantly, the haplodiploid genetic system found in the Hymenoptera allows for the production of biased sex ratios through the control of fertilisation (Hamilton, 1967; Werren, 1987). The (apparent) simplicity of this mechanism has no doubt helped the field develop in parasitoids, unlike vertebrate sex allocation where the mechanisms and the occurrence of facultative sex allocation itself are more controversial (e.g. West, Shuker & Sheldon, 2005).

Sex allocation theory rests on Fisher's (1930) principle and the extension by Hamilton (1967). Fisher's (1930) insight was that the rarer sex obtains a mating advantage, and so mothers should over-produce this sex, all other things being equal. This leads to frequency-dependent selection on sex ratio, favouring a numerical sex ratio of 0.5. However, if one sex is costlier to produce than the other, Fisher's principle predicts an investment ratio of 0.5, which leads to more of the cheaper sex being produced. Importantly, this frequency-dependent component of sex ratio selection will act regardless of what the optimum investment sex ratio is (i.e. the frequency-dependent effect holds even if the optimum ratio is not 0.5). Hamilton (1967) realised that reasons other than frequency-dependent mating success will contribute to parental fitness. He showed that if one sex was more beneficial to produce (or, to put it another way, less costly in net fitness terms), females should over-produce that sex, up to the point that there was a balancing mating advantage to being the rarer sex. There are a number of ways in which the costs and benefits of producing one sex *versus* another may vary. These can be grouped into two classes: (*i*) those that arise from localised interactions among kin (Hamilton, 1967; Clark, 1978; West *et al.*, 2005); (*ii*) those that arise directly from the relationship between offspring condition and fitness (the Trivers–Willard effect: Trivers & Willard, 1973).

If kin of one sex compete for mates, then that competition is costly for the mother, and so natural selection should favour mothers that over-produce the non-competing sex (Hamilton, 1967). For kin to interact and compete for mates greater than at random, populations have to be highly structured so that kin commonly interact. This means that the mating system is a key component of sex ratio selection (West & Sheldon, 2002). Hamilton (1967) termed this competition for mates among relatives local mate competition (LMC). Typically, related males (brothers, half-brothers and so on) will be competing for mates, and so female-biased sex ratios are predicted. This female bias reduces fraternal competition for mates and also increases the mates available for sons (Taylor, 1981). Clearly the mating systems of many gregarious and quasi-gregarious parasitoids, formed by the patchy distributions of their hosts, will lead to local mating patches and interactions among kin (we have already discussed the inbreeding that results from these local mating patches). Hamilton (1967) realised that organisms such as parasitoid wasps often had a life history in which LMC would favour female-biased sex ratios. Moreover, as more females contribute eggs to a given ‘patch’ of hosts, LMC (competition among relatives) will be ameliorated and less-biased sex ratios will be favoured. Given that our main focus is sexual selection it is worth emphasising that total competition for mates (as well as variance in male mating success) may increase even while local mate competition decreases. The sex ratio predictions central to LMC (female-biased sex ratios which become less biased with increasing numbers of unrelated individuals) have been repeatedly tested and verified in many parasitoids (Werren, 1980, 1983, 1984, 1987; Griffiths & Godfray, 1988; Godfray, 1994; King, 1996; West *et al.*, 2005; West, 2009). Additionally, there have been a number of important extensions to the basic LMC idea, where the level of LMC varies due to things like the extent of male dispersal to find additional matings (partial LMC: Hardy, 1994; West & Herre, 1998) or the extent of temporal or spatial overlap of different broods on a patch (asymmetric LMC: Shuker *et al.*, 2005, 2006). In *H. hebetor* for example, females are more likely to disperse and do so earlier than males, often prior to mating (perhaps to reduce inbreeding in this species which exhibits sl-CSD). This will increase the level of LMC in a patch (males will be competing over fewer virgin females that have not yet dispersed) and so females would be expected to produce a sex ratio biased more towards daughters than under basic LMC (Ode, Antolin & Strand, 1998). Generally, however the key insight of Hamilton's LMC theory remains the same: the optimal sex ratio depends on the extent to which related males compete for mates.

Of course, organisms do not only compete for mates, and LMC is really only a subset of a broader aspect of sex-allocation theory, namely local resource competition (LRC: Charnov, 1982). For instance, if female offspring compete for resources required for reproduction, male-biased sex ratios may be favoured by selection. The importance of LRC (outside of LMC) in parasitoids is not clear. If host availability is low then reproductive competition between daughters will limit reproductive success, although the extent to which females would come to compete might be limited under natural conditions. However, rather than biasing the sex ratio towards males, female parasitoid wasps could perhaps invest more in individual daughters (providing them with a competitive advantage). For instance, females of the gregarious parasitoid wasp *Goniozus nephantidis* Muesebeck (Bethylidae) lay smaller clutches (which produce larger females) when competition over hosts is high; this is adaptive as a size advantage allows daughters to guard hosts aggressively (Goubault, Mack & Hardy, 2007).

Another key concept in sex allocation is local resource enhancement (LRE) whereby individuals of one sex provide relatives (usually parents) with greater fitness returns, for instance through alloparental behaviour (e.g. in cooperative breeders: Emlen, Emlen & Levin, 1986). Again, the role if any of LRE in parasitoid sex allocation is not clear. For gregarious parasitoids, a bias towards the ‘least unhelpful’ sex may be more relevant. Asymmetric larval competition arises when the competition within a host influences the fitness of one offspring sex more than another, so a bias towards the sex that causes the least competitive effect may result [although Ode, Antolin & Strand (1996) found that despite the female bias in *H. hebetor*, female larvae represented stronger competitors; see also Godfray (1986) and Sykes *et al.* (1985)].

Finally, for solitary species the role of LRC or LMC in sex allocation may be limited (ignoring complications of superparasitism). Instead, these species tend to allocate sex according to host size, a form of condition-dependent sex allocation (Trivers & Willard, 1973). Typically, females gain more in terms of reproductive success from being large (Godfray, 1994). Since offspring size is often strongly associated with host resources (especially for solitary wasps), solitary female parasitoids tend to lay daughters on large hosts and sons on smaller hosts [meeting the predictions of the Trivers & Willard (1973) hypothesis; there is a very considerable literature on host-size-dependent sex allocation, see Charnov, 1982; Godfray, 1994; West & Sheldon, 2002; West, 2009]. Whilst resource availability may influence sex allocation in gregarious species as well, it appears that LMC appears to dominate sex allocation in those cases.

As is clear from the above, sex allocation has been subject to extensive study in parasitoid wasps, both due to the remarkable precision of adaptation it demonstrates and the economic value of manipulating the sex ratio (Ode & Hardy, 2008). Indeed, in terms of the reproductive biology of parasitoids, most research has focused on their economic importance as agents for biological control (Powell, 1986). In terms of biological control, a female-biased sex ratio is clearly highly desirable as it is females which are responsible for reducing the pest species' population size (Zhishan *et al.*, 2003).

## V. THE OCCURRENCE AND EVOLUTION OF POLYANDRY

Crucial to understanding mating systems and the patterns of sexual selection (and sexual conflict) that arise from them is the degree of female remating (with different males, i.e. true polyandry). Polyandrous females will spend more time ‘in’ the mating pool, influencing the operational sex ratio (OSR) and the extent of competition amongst males for fertilisations. For many insects, the prevailing wisdom has been that females get little apart from sperm from mating. However, in a landmark meta-analysis of 122 studies, Arnqvist & Nilsson (2000) found clear fitness benefits of polyandry to females across a wide range of insect species. The benefits of multiple mating to females have been subject to fairly extensive study. Females may gain direct benefits such as the replenishment of sperm supplies (Thornhill & Alcock, 1983; Siva-Jothy, 2000), access to resources or territories, or nuptial gift transfer (Fedorka & Mousseau, 2002). Indirect genetic benefits may also play a part in the evolution and maintenance of polyandry. Females can benefit through increased offspring viability and attractiveness, as envisaged by classic ‘good genes’ and ‘sexy sons’ models (Andersson, 1994; also see Section II.2^1994^ for a discussion of indirect processes of sexual selection in haplodiploids). Alternatively females may mate with several males to ensure genetic compatibility with at least one and/or promote genetic diversity of their offspring, which can be particularly beneficial in the event of environmental change (Andersson, 1994; Arnold & Duvall, 1994; Jennions & Petrie, 2000; Bernasconi & Keller, 2001; Konior, Radwan & Kolodziejczyk, 2001). Females may also mate with multiple males simply to escape the costs of male harassment (convenience polyandry; Thornhill & Alcock, 1983).

Despite the apparent benefits, parasitoids are one insect taxon where polyandry is not prevalent (Allen, Kazmer & Luck, 1994; Jacob & Boivin, 2005). Ridley (1993) found that around 70% of parasitoid wasp species are monandrous. But is monandry in the parasitoid wasps ‘true’ (or strict) monandry (i.e. a complete and irreversible loss of receptivity after an initial mating) or does it result from a change in receptivity combined with key aspects of the mating ecology, for instance post-mating dispersal (‘effective’ monandry)? For instance, there is some evidence that female *Nasonia vitripennis* are occasionally multiply mated in the wild (Grillenberger *et al.*, 2008), suggesting that effective monandry may be the norm here. Indeed, ‘true’ monandry may be rare in insects. To give just one example, female *Aedes aegypti* L. (Culcidae) mosquitoes were long assumed to be monandrous based on laboratory observations, whereas in the wild 14% of females were shown to have mated with a second male (through labelling of sperm using stable isotopes; Helinski *et al.*, 2012). It seems likely then that true monandry will be rarer in parastioids than we currently think.

In recent years understanding the evolution of polyandry has gained prominence in debates about sexual selection and sexual conflict (Pizzari & Wedell, 2013). A common problem for many studies that look at the costs and benefits of polyandry though is that we typically study species that are already polyandrous. This risks us conflating selection that maintains polyandry with the selection that might have led to its original evolution. As such, parasitoids may prove useful as study organisms given their common but not strict patterns of monandry. For instance, van den Assem & Jachmann (1999) were the first to observe an increase in polyandry in populations of *Nasonia vitripennis* that had been maintained in the laboratory (polyandry appears to be rare but not absent in the wild; see above). Subsequent studies have confirmed that the probability of remating in this species increases with time spent under mass culture conditions (Burton-Chellew *et al.*, *a*^2007^; Grillenberger *et al.*, 2008) and polyandry has both an additive and a non-additive genetic component (Shuker *et al.*, 2007). However, laboratory selection does not affect all species of *Nasonia* equally, as in a separate study *Nasonia longicornis* were less likely to remate than *N. vitripennis* (Leonard & Boake, 2008).

When considering the benefits of polyandry in parasitoids, Ridley (1993) found support for a sibling competition explanation, based on the finding that it occurs more frequently in gregarious species. Ridley (1993) postulated that polyandry reduces competition between siblings when they compete together for host resources, by reducing the degree of relatedness among brood-mates. By contrast, Godfray (1994) considered a sperm-depletion explanation to be relevant to gregarious species, as it is important for females to obtain sufficient sperm to produce a female-biased sex ratio due to local mate competition (LMC). Solitary species on the other hand will suffer less from being unmated as their sons will experience little or no fraternal competition over mates. Additionally, males in gregarious species are more likely to become sperm depleted in the first place due to the female-biased sex ratio and the rapid sequential matings they perform (Hardy *et al.*, 2005).

Several examples from the parasitoids highlight how sperm depletion may or may not favour polyandry. For instance, female *Cotesia congregata* Say (Braconidae), a gregarious species, produced more daughters when provided with multiple mates (Freeman & Kester, 1999). Additionally, *Cephalanomia hyalinipennis* (also gregarious) females may choose to remate, and thus previously sperm-depleted females resume daughter production (Pérez-Lachaud, 2010). However, King & Bressac (2010) found that in the generally monandrous solitary parasitoid wasp *Spalangia endius* Walker (Pteromalidae), females did not benefit from sperm accumulated from experimentally induced additional matings, despite experiencing a rapid decline in daughter production later in life. We will discuss sperm depletion in more detail in Section VI.5*b*^1989^.

## VI. Sexual Selection in Parasitoid Wasps

### (1) Defining sexual selection

Sexual selection arises from variation in competition for mates, or competition for fertilisations more generally (Darwin, 1859, 1871; Andersson, 1994; Shuker, 2010). Competition for mates can occur through a variety of direct contests, or indirectly through passively or actively attracting members of the opposite sex. Following Darwin (1859, 1871), sexual selection has traditionally been broken down into intra-sexual contest competition (male–male or female–female competition) and inter-sexual mate choice. The former has never been controversial, whilst mate choice has long been associated with one form of controversy or another (see, for instance, Halliday, 1983; Bradbury & Andersson, 1987; Cronin, 1992; Andersson, 1994). What is less controversial about mate choice is its definition: following Halliday (1983) among others, we will consider mate choice as occurring when any phenotype (behavioural, morphological, physiological) in one sex leads to non-random success in achieving matings in the other sex. Also uncontroversial since the insights of Parker (1970) is that sexual selection can occur after insemination, when ejaculates from multiple males may compete to fertilise eggs, and that females may bias this competition through cryptic female choice (Eberhard, 1996; Simmons, 2001).

In recent years though, the very definition of sexual selection has come under some scrutiny (Shuker, 2010). Of most relevance here has been the suggestion that female–female competition for resources associated with reproduction should also be included under the banner of sexual selection, not just competition for mates (e.g. Clutton-Brock, 2007, 2009; Rosvall, 2011). One problem with this suggestion is that virtually all resource competition that influences a female's inclusive fitness has to influence her reproductive success and so nearly all natural selection becomes subsumed by sexual selection (or *vice versa* depending on your point of view; see Shuker, 2010, for a brief discussion). Whilst introduced perhaps with vertebrates primarily in mind, there are potential examples among parasitoid wasps. For instance, competition over hosts on which to oviposit is near-ubiquitous for female parasitoids and the extent varies with the level of gregariousness of the species in question (Godfray, 1994). Solitary species are likely to experience greater competition over hosts as multiple females cannot superparasitize a single host like gregarious species. Lawrence (1981) found that in the solitary braconid *Biosteres longicaudatus* (Ashmead), increasing parasitoid density resulted in increased female–female aggression, whilst introducing more hosts served to alleviate the aggression to some extent. Gregarious species can still gain some fitness from laying eggs on an already parasitised host (superparasitism), although offspring will often be small and of lower fitness (Godfray, 1994). Nonetheless, there can be considerable female–female competition for hosts in the gregarious *Goniozus nephantidis* (Goubault *et al.*, 2007) and losers have an intriguing adaptation by which they emit a puff of pheromone that appears to either signal submission or actively disorientate the winner, allowing the loser to flee the scene (analysed in wonderful detail using real-time chemical analyses by Goubault *et al.*, 2006). Even in the gregarious *Nasonia vitripennis*, when several females that have not had access to hosts for 2 days are presented together with a host, loss of antennae and legs can result from the interactions between ovipositing females (D. M. Shuker, personal observations). However, as interesting as these observations are, we do not consider these forms of female–female resource competition as generating sexual selection, as they do not concern competition for mates.

### (2) The operational sex ratio and related measures of mating opportunities

The operational sex ratio (OSR; ratio of males to females in the population that are currently reproductively capable) is inexorably linked to both sex allocation and the mating system (Emlen & Oring, 1977; Davies, 1991; Parker & Simmons, 1996; Kokko & Jennions, 2008). The relative availability of males and females in the mating pool at any given time (and the ‘time-out’ each sex has following a sexual encounter: Parker & Simmons, 1996) is captured by the OSR (Emlen & Oring, 1977). For example, if females are rarely in the mating pool, there should be strong selection on males to find receptive mates and guard them. Despite (or perhaps because of) the apparently straightforward logic, there remain very few convincing experimental tests of the role of OSR in mating systems, and in terms of the resulting patterns of sexual selection. Moreover, the links between OSR, mating systems and sexual selection remain controversial (Kokko, Klug & Jennions, 2012). Alternative measures to capture the essence of mating systems have been proposed, including the potential reproductive rate (PRR: Clutton-Brock & Vincent, 1991; Clutton-Brock & Parker, 1992), or the opportunity for sexual selection (Shuster & Wade, 2003). While a full discussion is beyond the scope of this review, the abundance of information on sex allocation in parasitoids offers opportunities for exploring how OSR interacts with processes of mate competition.

### (3) Sexual dimorphism: a role for sexual selection?

**Figure 2:**
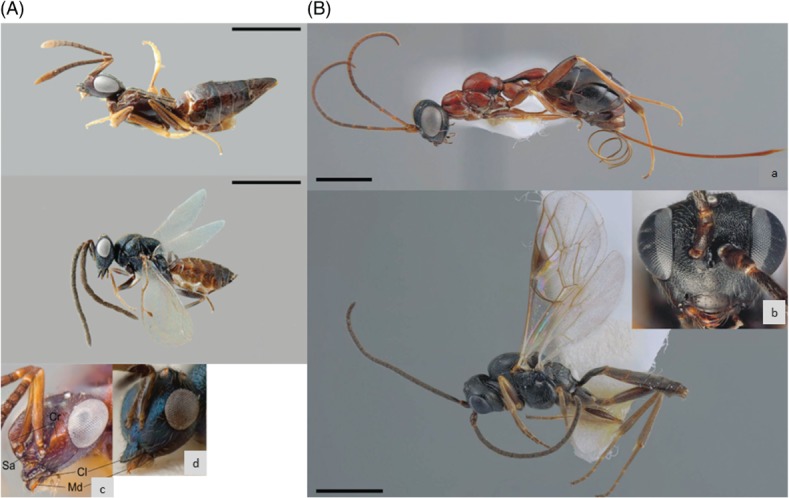
(A) *Calymmochilus dispar,* brachypterous female in lateral view (a), head (c); male in lateral view (b), head (d). Abbreviations: Cl: clypeus; Cr: crest; Sa: supraclypeal area; Md: mandible. Scale bar = 1 mm. (B) *Gelis apterus*, apterous female in lateral view (a), male in lateral view (b), and male head in front view (c). Scale bar = 1 mm (from Korenko *et al.*, 2013).

Sexual dimorphisms evolve for a variety of reasons, including different ecological roles of males and females, or through competition for mates (Fairbairn, Blanckenhorn & Székely, 2007). Females are typically the larger sex in the parasitoid wasps and amongst the Hymenoptera generally, probably as the fitness advantage of size is greater in terms of female fecundity than male mating and fertilisation success (Hurlbutt, 1987; Godfray, 1994; for a thorough review of sexual dimorphism in the Hymenoptera see Stubblefield & Seger, 1994). Many species of parasitoid wasp exhibit sexual dimorphism in traits other than size, and the importance of natural *versus* sexual selection in shaping these traits remains unknown. For instance, in *Spalangia dozieri* the males have conspicuously enlarged hind legs (Fig. 1; Gibson & Reigada, 2009). The leg morphology is suggestive of a function involving grasping and holding. Gibson & Reigada (2009) suggest that this enables male phoresy or functions in sexual behaviour, for instance in male–male contests or for grasping females during mating. Similarly, two species of sexually dimorphic wasps which parasitise the ant-eating spider [*Zodarion styliferum* (Simon) (Zodariidae)] have recently been discovered. *Calymmochilus dispar* Boucek & Andriescu (Eupelmidae) and *Gelis apterus* Pontoppidan (Ichneumonidae) both exhibit fairly striking sexual dimorphism in colour and wing dimorphism (with brachypterous and apterous females respectively; see Fig. 2; Korenko *et al.*, 2013). Females may also be the more ornamented sex. For instance, females of *Comperiella bifascia* (Encyrtidae, Howard) display curled and conspicuously marked wings which contrast drastically with the understated apperance of the male (Compere, 1926). As in *S. dozieri*, the repertoire of sexual behaviour which all these species display has yet to be characterised but such examples will hopefully stimulate interest in sexual selection in species such as these.

### (4) Pre-copulatory intra-sexual competition

Pre-copulatory intra-sexual competition has received surprisingly little attention in parasitoids, despite its importance in sexual selection in animals more generally (Andersson, 1994) and the fact that it is likely to be common in parasitioids as well. Here we will consider what mechanisms of intra-sexual competition have been explored and what traits may have evolved in response.

#### (a) Protandry

**Figure 3:**
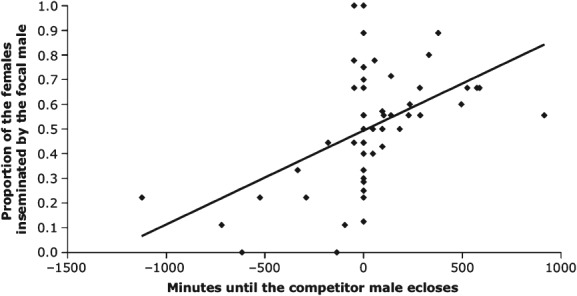
Protandry in *Nasonia vitripennis* is under sexual selection as indicated by the positive relationship between the difference in emergence time of focal/competitor males and the focal males insemination success (data from Moynihan & Shuker, 2011).

Protandry (when males emerge before females) is widespread in the parasitoids and males of many species may benefit from early emergence by intercepting and inseminating females before they have a chance to disperse (Godfray, 1994). From life-history theory, one might predict that males face a trade-off between adult body size and development time (e.g. Stearns, 1992), such that large males could be at a physical competitive advantage, but smaller males may benefit from achieving early matings if they develop faster. Some examples of the interaction between protandry and mating success have been documented in parasitoid wasps (see Table 2003). In *Cardiochiles nigriceps* Viereck (Braconidae) for instance, early emergence is advantageous to males (Hirose & Vinson, 1988). This study did not, however, consider the interaction between body size and protandry. Moynihan & Shuker (2011) on the other hand did consider this trade off in *Nasonia vitripennis*. They showed a sexual selection benefit to protandry (see Fig. 3), but found no evidence that small males benefit. Instead, large males emerged earlier than smaller males. They also found that the benefit of early emergence outweighed any competitive advantage that large body size might have offered. These studies did not, however, quantify the mortality risk prior to female emergence or the level of male competition on which the optimal relative emergence time will depend. If mortality risk is high it may be better to emerge later and risk missing matings with early-emerging females. Likewise if male–male competition is lower for later emerging males, a wider range of relative emergence times will be expected (Godfray, 1994). In *Melittobia australica* Girault (Eulophidae) however, earlier emerging males are at a competitive advantage despite the high mortality risk. In this species, the mortality risk occurs as a result of lethal male competition, with earlier emerging males typically killing all younger males (Abe *et al.*, 2003; see above).

A recent study by Macedo *et al.* (*a*)^2013^ considered protandry both within and between cohorts of emerging *Allorhogas dyspistus* Marsh (Braconidae) wasps. These quasi-gregarious braconids are gallers and parasitise the seeds of *Pithecellobium tortum*, reproducing throughout the year with overlapping generations. Females mate only once, whereas males mate repeatedly across their life. *A. dyspistus* shows the protandry typical of many quasi-gregarious parasitoid wasps, but the extent of this protandry also varies seasonally because females produce a male-biased sex ratio at the beginning of the reproductive period, whilst later sex ratios become progressively female biased. The adaptive value for this behaviour was considered by West & Godfray (1997). The basic idea is that with over-lapping generations, during periods of high recruitment producing males early is beneficial. West & Godfray (1997) demonstrated how the optimal sex ratio depends on female reproductive strategy, with the bias being less extreme if females mate throughout their life (see Werren & Taylor, 1984). This example illustrates how female reproductive strategy and reproductive competition (which will increase at times of high recruitment, i.e. when reproductive output and survival to adulthood is high) dictates sex allocation and how this affects the OSR, feeding back into the mating system and influencing sexual selection on male protandry.

#### (b) Contests and scrambles

Competition for mates may be more direct, with individuals fighting or scrambling for access to mates. Contests may select for weaponry, and scrambles may select for adaptations such as mate-searching ability and rapid copulations. For instance, in *Melittobia australica* males engage in aggressive contests where the majority of competitors are killed; males possess mandibles which they use only for fighting (they do not feed after emergence; Abe, Kamimura & Shimada, 2005). Innocent *et al.* (2011) found that *M. australica* males did not adjust the frequency and intensity of fights with variation in the value of females as mates (i.e. the greater value of virgin than mated females) or variation in the relatedness of competitors. Fight intensity did, however, increase with higher competitor densities. The authors suggest that the short lifespan of males combined with the limited mating opportunities means that they should fight whenever they encounter a competitor without assessing the value of the resource. These results also suggest that, as in other non-social insects, *M. australica* show no ability to discriminate kin. One of many possible explanations for this is due to the local mating structure in this species. As competition becomes more local, the competition between relatives increases and the kin-selection advantage of altruism is reduced (West, Pen & Griffin, 2002). In addition to kin discrimination, this system also relates to sex allocation: female *M. australica* do not allocate sex in response to superparasitism, instead they consistently produce males at a low rate, which will reduce the mortality caused by fighting between brothers (Abe *et al.*, 2005).

Large body size is often assumed to be beneficial in competitive encounters, indeed in solitary *Eurytoma* sp wasps large size is advantageous in encounters at female emergence sites (Macedo *et al.*, 2013*b*). However, large size is often not the most important trait. Part of this may be because competition is as much by scrambles for females as it is by contests. For instance, Cheng *et al.* (2003) found no effect of size on success in male contests in *Cephalonomia tarsalis* Ashmead (Bethylidae). Amongst the winners, small males exhibited more competitive behaviours than large males. However, losing males were generally sucessful in engaging in a subsequent mating with a female. Smaller male *Lariophagus distinguendus* Förster (Pteromalidae) were found to be disadvantged in terms of reproductive success compared to larger conspecifics to some extent, but male size was not associated with courtship behaviour or mating performance when males were provided with five females simultaneously, and size did not dictate which male mated first in a two-male, one-female competitive situation (van den Assem, van Iersel & Los-Den Hartogh, 1989). In *Nasonia vitripennis* there are reports of males guarding exit holes and monopolising matings, and so one might expect large males to be advantaged under such circumstances. However, Burton-Chellew *et al.* (*b*)^2007^ found that male size did not impact mating success with or without the presence of a competitor. On the other hand, a recent study by Blaul & Ruther (2012) showed that large males of *N. vitripennis* were at a competitive advantage in terms of inter-sexual competition: they were more attractive to females at a distance. But, when direct intra-sexual competition is taken into consideration there was no net effect of size on mating success. Instead, small males are at a competitive advantage during mate acquisition, recognising females significantly faster than large males in both competitive and non-competitive situations; when in direct competition with a larger male, the small male was significantly more likely to be the first to mount. Clearly intra- and inter-sexual selection on body size are often linked; we will revisit mate choice based on size more explicitly in Section VI.5*c*^1999^.

#### (c) Alternative mating tactics

Competition for mates often leads to the adoption of alternative mating tactics (Oliveira, Taborsky & Brockmann, 2008). Alternative mating tactics is a sizeable field in its own right, and we here introduce some examples currently known about in parasitoids and related groups. Perhaps the most straightforward alternative mating tactic (AMT) is to avoid potentially costly courtship or territory-holding behaviours. In *Nasonia vitripennis* (van den Assem & Beukeboom, 2004) and in *Cotesia rubecula* Marshall (Braconidae) (Field & Keller, 1993) males have been observed to ‘sneak’ copulations without courting by taking advantage of female receptivity elicited by a more honest male (although the possibility that this behaviour represents a distinct tactic rather than the outcome of a mating scramble remains to be tested). On the other hand, AMTs may involve the evolution of marked polymorphisms in morphology and behaviour. For instance, it is well established that many species of fig wasp exhibit polymorphism in male mating strategies and associated morphological traits such body size and weaponry (Hamilton, 1979). Large ‘fighting’ males possess heavy armour and large mandibles and are often wingless, competing intensely to mate with females inside the fruit. ‘Sneaker’ males on the other hand are much smaller and often possess wings allowing them to mate with females after dispersal (Bean & Cook, 2001; Moore, Pienaar & Greeff, 2004). However, male morphs are not always so conspicuous, and cryptic male morphs have been uncovered in fig wasps using rigorous examination of morphology and allometry (Cook & Bean, 2006).

A recent study has found evidence for AMTs in a solitary (non-gregarious) *Eurytoma* (Eurytomidae, Illiger) wasp. Macedo *et al.* (*b*)^2013^ found that variance in body size was higher for males, while the majority of medium-to-large individuals were female. Males were typically either small or very large, and large males were also more likely to engage in fights at female emergence sites. This suggests that large and small males may represent physically and behaviourally distinct morphs with small males adopting a satellite strategy. If AMTs such as this are found to exist in parasitoid wasps more generally, the underlying proximate mechanisms could be a particularly fruitful avenue for further investigation. For example, condition-dependent sex allocation (Trivers & Willard, 1973; see Section IV^1994^) could permit maternal control of offspring body size by laying mostly males on large/high-quality and small/low-quality hosts, and females on intermediate size/quality hosts. Females would then be able to enhance their own fitness not just by altering the sex ratio they produce, but also by influencing the strategies their sons adopt, which may be frequency dependent and/or vary with maternal condition and environmental factors.

One other intriguing possible AMT has been revealed in *Lariophagus distinguendus*, a parasitoid of beetle larvae within cereal grains (Steiner, Steidle & Ruther, 2005). Male *L*. *distinguendus* are generally protandrous, emerging before females. Female pupae release a pheromone which attracts males, causing them to stop and even start courting female pupae. However, developing male pupae produce the same pheromone, which again elicits adult male arrestment and wing-fanning behaviour. Steiner, Henrich & Ruther (2008) suggest that this is a form of intra-specific sexual mimicry. Late-emerging males are inherently disadvantaged compared to early emergers, since females of this species mate monandrously and so late emergers are less likely to encounter receptive females. By producing this pheromone however (which they appear actively to decompose within 32 h of emergence) they may be able to distract already-emerged males and increase their own chances of mating with a receptive female upon emergence; this possibility remains to be tested empirically. Males of *Cotesia rubecula* may also occasionally mimic females. By adopting the female receptivity posture they can decieve a second male into attempting copulation, distracting them in order to attain a mating with a nearby female (Field & Keller, 1993).

In summary, there are some tantalising examples of AMTs in parasitoids, but amongst the parasitoids and related taxa, only fig wasps have really been subjected to rigorous behavioural and morphological testing to determine the existence of different male morphs; much more may remain to be discovered.

### (5) Pre-copulatory mate choice

Much of the work on the evolution of mate choice has been concerned with the benefits that accrue to the choosing sex (often but not always females: Bateson, 1983; Andersson, 1994; Bonduriansky, 2001). Detailed analysis of the benefits of mate choice are scarce in parasitoids though. In some cases detailed below, direct benefits are clear (in particular the provision of sufficient sperm), and there is also an excellent system for the study of genetic compatibility *via* inbreeding avoidance (when sex determination is mediated by CSD), but these are exceptions. One issue that needs to be remembered is that many aspects of life history and fitness will be host dependent, perhaps limiting the extent to which traits can reflect overall genetic ‘quality’. In other words, we might expect common genotype-by-environment (G × E) interactions for fitness in parasitoids. This may be particularly true for koinobionts (where eggs are laid when hosts are still growing) *versus* idiobionts (where eggs are laid once final host size has been determined). In idiobionts the ovipositing female can accurately assess host quality (and can use this information in sex allocation decisions; Ode & Heinz, 2002) and so variation in body size may be more relevant as a signal of genetic quality. Indeed, comparisons of these two kinds of parasitoids with respect to patterns of mate choice (for body size perhaps) and fitness variation would be an interesting and novel test of the role of genetic quality and G × E interactions in sexual selection (Ingleby, Hunt & Hosken, 2010).

#### (a) Opportunities for mate choice?

Due to the local mating structures often observed in parasitoids, females may have few males to choose from in the mating pool, mating mostly with brothers who emerge close-by, both spatially and temporally (van den Assem, 1986). This may explain why in some species such as *Nasonia vitripennis* virgin females appear to be fairly indiscriminate in mating, typically accepting the first male they encounter, and also why there have been so few studies of male and female mate preferences in parasitoids. Those there have been have focused largely on body size and mating history (Table 2003). Many of these studies have yielded negative results, but thus far relatively few species and traits have been tested. Moreover, some species contain morphologically unusual males. For example, what is the significance of the conspicuously ornamented mid-legs in *Mesopolobus* sp Westwood (Pteromalidae)? Some males of this genus have a striped yellow/orange mid-tibia with a black knob which they pass over the females eyes during courtship (Askew, 1971; van den Assem, 1974), suggesting a possible function in female choice (Godfray, 1994).

#### (b) Mate choice and sperm depletion

**Figure 4:**
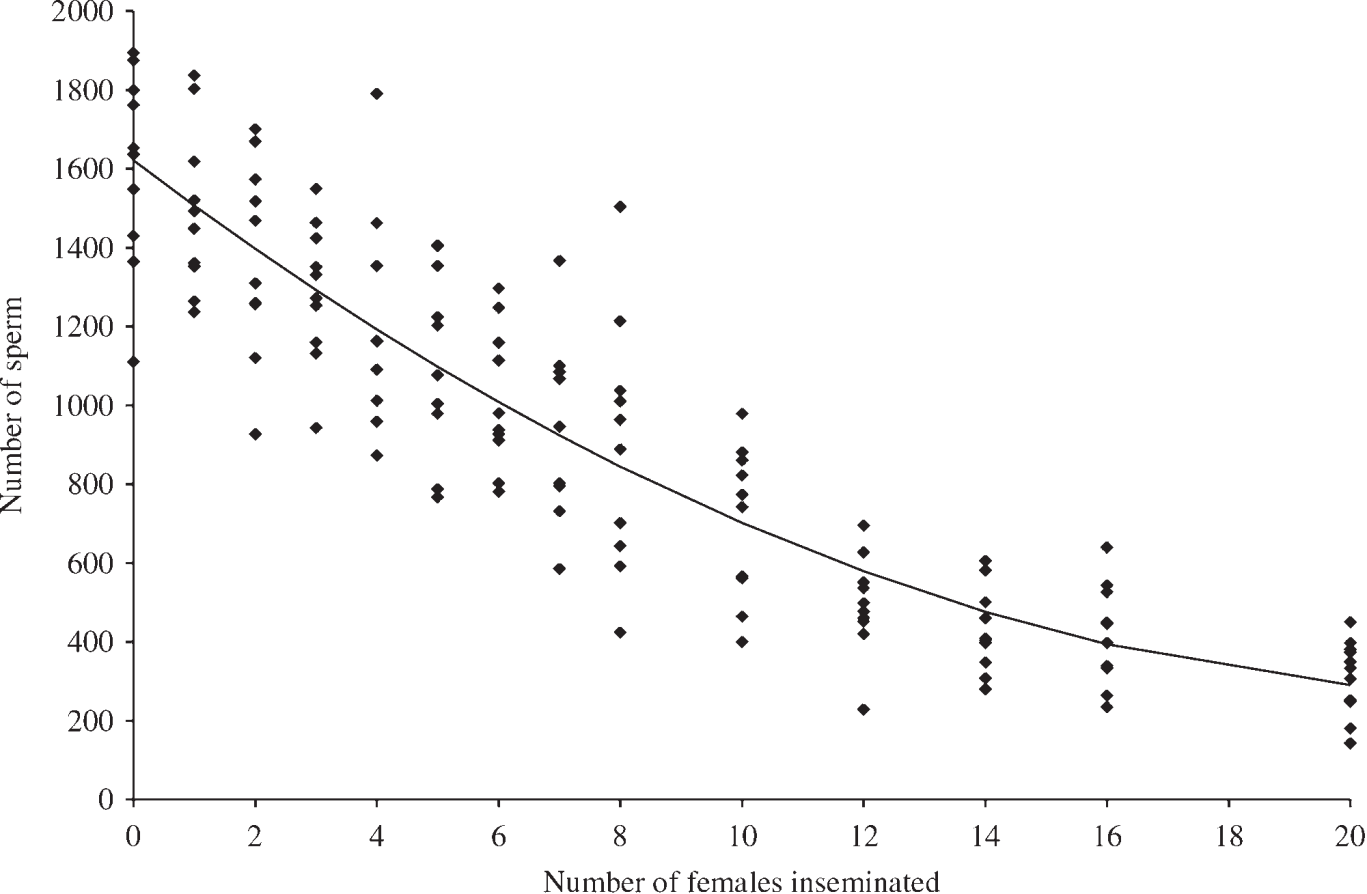
Number of sperm remaining in seminal vesicles of male *Trichogramma euproctidis* (*evanescens*) after successive copulations. Each point corresponds to the quantity of sperm present in both seminal vesicles of one male (data from Damiens & Boivin, 2005).

The risk of sperm depletion is a common reproductive problem throughout the parasitic Hymenoptera [Godfray, 1994; *Spalangia cameroni*, King, 2000; *Trichogramma euproctidis* Girault (Trichogrammatidae) (previously thought to be two separate species, *T. evanecens* Westwood and *T. turkestanica* Meyer), Damiens & Boivin, 2005; *Lariophagus distinguendus*, Steiner *et al.*, 2008; *Cephalanomia hyalinipennis* Ashmead (Bethylidae), Pérez-Lachaud, 2010; *Pachycrepoideus vindemminae* Rondani (Pteromalidae), Nadel & Luck, 1985]. Many male parasitoids are prospermatogenic (i.e. they emerge with their full complement of spermatozoa; Damiens & Boivin, 2005), and some species are limited in their ability to fertilise females (see Fig. 4; *T. euproctidis*, Damiens & Boivin, 2005, 2006; *C. hyalinipennis*, Pérez-Lachaud, 2010; *S.cameroni*, King, 2000; but not *Pteromalus venustus*, Tepedino, 1993). Despite this, males have been found to continue to attract and mate with females (*T. euproctidis*, Damiens & Boivin, 2006). For instance, *Nasonia vitripennis* males are able to mate with hundreds of females (Barrass, 1961) but only inseminate a small proportion during the earlier copulations (van den Assem, 1986).

**Figure 5:**
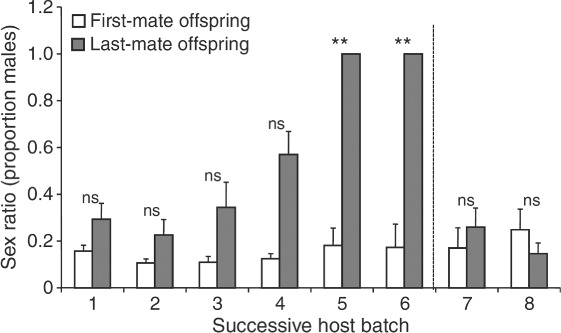
Variation in the mean (+S.E.M) sex ratio of offspring of the first (N = 13) and last (seventh, N = 13) female to mate with the same *Cephalonomia hyalinipennis* male (host batches 1–6) and offspring sex ratio of the same females following second mating with a different male (batches 7 and 8). Data from Pérez-Lachaud (2010). ** indicates a significant difference (*P* < 0.008); ns, no significant difference.

Mating with a sperm-depleted male can negatively impact female fitness. Females lacking sufficient sperm (‘constrained’ females in the parasitoid literature) will be unable to allocate their resources optimally between sons and daughters (Godfray, 1990; Ode, Antolin & Strand, 1997; Henter, 2004; Ruther *et al.*, 2009). This has been documented in females of *Aphidus ervi* Haliday (Braconidae) and *Cephalanomia hyalinipennis* (Fig. 5, Henter, 2004; Damiens & Boivin, 2006; He & Wang, 2008; King & Bressac, 2010; Pérez-Lachaud, 2010). Additionally, a study by Ode *et al.* (1997) implied that sperm depletion is a biologically relevant phenomenon in *Habrobracon hebetor*, as 20% of wild females had no sperm in their spermatheca and on providing wild females with hosts daughter production rapidly ceased.

**Figure 6:**
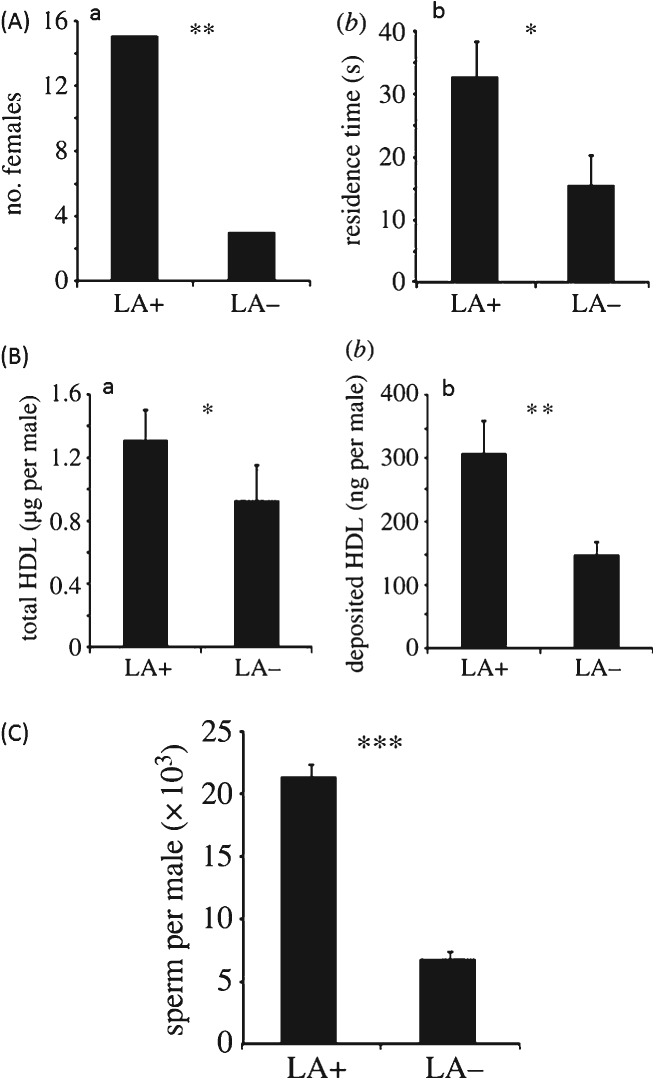
(A) Response of virgin *Nasonia vitripennis* females in an olfactometer bioassay to pheromone deposits released by males from hosts supplemented with linoleic acid (LA+, N = 23) and not supplemented (LA−, N = 20). First choice for (a) and mean + S.E.M. residence time in (b) pheromone-deposit-marked cavities within an observation time of 5 min. (B) Total (4R, 5R) and (4R, 5S)-5-hydroxy-4-decanolides (HDL) amounts (mean + S.E.M) (a) extracted from the abdomen and (b) deposited within an observation time of 10 min by 2-day-old *N. vitripennis* males from LA+ and LA− hosts, respectively. (C) Mean + S.E.M. sperm number counted in the seminal vesicles of individual *N. vitripennis* males from LA+ and LA− hosts. Data from Blaul & Ruther (2011). ***P < 0.001; **P < 0.01; *P < 0.05.

Sheldon (1994) proposed that female birds seek extra-pair copulations in order to insure against functional infertility of their mates. The ‘fertility-insurance’ hypothesis may be applied to parasitoid wasps when female fitness is limited by constrained daughter production from mating with a sperm-depleted male [akin to Godfray's (1994) sperm-depletion explanation for the higher incidence of polyandry in gregarious parasitoids, see Section V^1995^]. Alongside female remating we should expect traits to evolve that permit female discrimination of sperm-limited or sperm-depleted males (Luck & Joly, 2005). King & Fischer (2010) found that female *Spalangia endius* exhibit a significant preference for virgin over mated males, however this effect was not entirely based on female choice as virgin males were also quicker to attempt copulation. Furthermore, King (2000) found that females could not discriminate between sperm-depleted and non-depleted males in *S. endius*. In *Nasonia vitripennis,* however, females appear to be able to detect male sperm load, showing a preference for sex pheromones produced by males with more sperm available (Ruther *et al.*, 2009). This preference is based on larger pheromone titres, as sexually immature and sperm-depleted males produce lower pheromone titres (Ruther *et al.*, 2007) and larger males with greater sperm resources secrete more pheromone (Steiner & Ruther, 2009; Blaul & Ruther, 2012). In addition to the aforementioned findings, Blaul & Ruther (2011) found a female preference for males from hosts who had been supplemented with linoleic acid [which is a precursor to the male sex pheromone (4R,5R)- and (4R,5S)-5-hydroxy-4-decanolides (HDLs)] (Fig. 6A). These males not only produced more HDL (Fig. 6B) but also three times more sperm than non-supplemented males (Fig. 6C).

Despite the benefits of avoiding sperm-depleted males, females do not always respond to sperm depletion. For instance, in one study with *Habrobracon hebetor*, over 60% of females ran out of sperm and were constrained to produce sons, but only 3 out of 64 remated (Ode *et al.*, 1997). Nor did Steiner *et al.* (2008) find any effect of mating status of the partner male (sperm-depleted/non-depleted) on female remating probability in *Lariophagus distinguendus* (a gregarious species), even though daughter production was reduced when females mated with sperm-depleted males. Similarly, Jacob & Boivin (2005) found that in the facultatively gregarious parasitoid wasp *Trichogramma euproctidis,* polyandry is the norm. They went on to investigate the costs and benefits of remating and found no longevity cost of refusing to mate, implying that polyandry does not occur due to the need to escape male harassment (convenience polyandry). Additionally, twice-mated females had increased longevity compared to monandrous females, but only in the presence of hosts. There is therefore a benefit of polyandry to lifetime reproductive success in *T. euproctidis*, although the proximate mechanism has not been elucidated, but in this case it does not necessarily relate to sperm accumulation as females which remated did not store additional sperm. Females which mated first with a sperm-depleted male and then with a virgin male did, however, benefit from additional sperm storage (although they must mate with three additional males to acquire the same amount of sperm stored by a female mated once to a virgin male; the reason for this reduced sperm storage is unclear: Damiens & Boivin, 2006).

In summary, sperm depletion and its limiting effect on female fitness should select for traits allowing females to overcome it, including both remating and choosing to mate with reproductively competent males, but clearly that is not always the case in the parasitoids studied thus far. Understanding why would be a major advance in our understanding of parasitoid reproductive biology. Becoming sperm limited will clearly also influence optimal male mating strategies, and economic ejaculate theory predicts that males will be more fastidious in their choice of mates when they have limited resources to invest (Engqvist & Sauer, 2001; Parker & Pizzari, 2010). For instance, Martel, Damiens & Boivin (*a*)^2008^ found that nutritionally deprived males of *Trichogramma euproctidis* exhibited more extreme preferences for virgin females than non-deprived males. We will consider the effects of sperm depletion on males further in Section VI.6*d*^2001^.

#### (c) Mate choice and body size

Often larger individuals will represent higher quality mates; as described in the previous section, large males in *Nasonia vitripennis* had larger sperm reserves and were preferred mates based on their pheromone profiles (Ruther *et al.*, 2007, 2009; Steiner & Ruther, 2009). In several other species larger males have a greater insemination capacity [*Habrobracon hebetor*, Ode, Antolin & Strand, 1995; *Colpoclypeus florus* Walker (Eulophidae), Dijkstra, 1986; two out of four species of Opiinae (Braconidae), Ramadan, Wong & Wong, 1991] and larger females are often more fecund (Hurlbutt, 1987; Godfray, 1994). Whether or not body size *per se* is under inter- or intra-sexual selection, will depend largely on the G × E interactions discussed in the introduction to Section VI.5^2004^. Female and male mate choice with respect to size varies across parasitoids (Table 2003) and the patterns could prove useful in comparative analyses of sexual selection on body size. In *H. hebetor*, females are more likely to mate with large males (Antolin, Ode & Strand, 1995) and smaller males take longer to induce receptivity and fertilise fewer females in their lifetime in *Trichogramma euproctidis* (Boivin & Lagacé, 1999). Additionally, Joyce *et al.* (2009) found a significant female preference for large males compared to small males in a simultaneous choice test in the solitary parasitoid *Cotesia marginiventris* Cresson (Braconidae); large and small males were equally likely to attempt copulation first but females more frequently accepted large males. By contrast, females of the closely related, gregarious *Cotesia flavipes* Cameron (Braconidae) did not exhibit any preference for large males; in *C. flavipes* males were on average larger than females and almost all attempted copulations were sucessful (Joyce *et al.*, 2009). This, combined with the strong female bias (broods consist of 80% females) and the high incidence of sibmating, might contribute to the lack of any preference in *C. flavipes*. Males may be a limiting resource and so females do not benefit from choosiness among broods that are often kin. With regards to male mate choice for female body size, Joyce *et al.* (2009) did not find any male preference in *C. marginiventris* but males of *C. flavipes* showed a preference for smaller females. The reasons for the latter finding were, however, not discussed. This, combined with the sparsity of studies which consider male mate choice (despite the clear fecundity advantages that would come with inseminating a large female) may reflect the underlying preconception (which has been subject to increasing challenge) that females are choosy and males are indiscriminate when it comes to mating.

#### (d) Mate choice and courtship displays

A variety of behavioural cues in the form of courtship displays may of course form the basis for mate choice (Andersson, 1994). During courtship, male *Nasonia vitripennis* mount females and position their mouthparts over the female's head and antennae, before beginning to perform a series of ‘head-nod’ behaviours, releasing a pheromone as they do so (van den Assem & Visser, 1976; van den Assem *et al.*, 1980). It seems that the olfactory component of head-nodding is more important than the tactile aspect as preventing head-nodding did not influence female acceptance rates, but glueing the mouthparts shut rendered males unable to elicit receptivity unless the pair were exposed to air passed over normal courting males (van den Assem *et al.*, 1980). *Nasonia vitripennis* males also perform wing-fanning movements during courtship (van den Assem & Putters, 1980) and there may also be acoustic signals produced. Recently Danci *et al.* (2010) also found evidence that male wing-fanning induces reply signals from females which help males to detect them in *Glyptapanteles flavicoxis* Marsh (Braconidae). In *Nasonia*, intra-specific variation in these aspects of courtship has received rather little attention (but see Peire-Morais *et al.*, 2003), with little evidence of pre-copulatory mate choice associated with courtship. However, these courtship displays certainly play a part in inter-specific mate choice in this genus (Beukeboom & van den Assem, 2001, 2002; van den Assem & Beukeboom, 2004) and may prove to be involved in intra-specific mate choice, for instance as in *Lariophagus distinguendus*; a recent study by Benelli *et al.* (2013) revealed that in this pteromalid females use fanning frequency as an indicator of male quality, and higher frequency wing-fanning was more likely to result in a sucessful mating.

#### (e) Mate choice and inbreeding

As we saw earlier, complementary sex determination (CSD) means that inbreeding can be extremely costly in many parasitoids, with the production of low-fitness diploid male offspring the result. Outbreeding in species with CSD therefore represents an interesting example of mate choice for genetically compatible mates (Zeh & Zeh, 1996, 1997; Tregenza & Wedell, 2000) and may prove useful for comparative analyses of the indirect benefits of mate choice (we would expect fewer indirect genetic benefits of remating and mate choice in obligate inbreeding species). Mechanisms that lead to non-random mating with respect to kinship can vary (Heimpel & de Boer, 2008). For instance, *Cotesia glomerata* L. (Braconidae) is a gregarious braconid with sl-CSD. Ruf, Dorn & Mazzi (2011) suggested that inbreeding avoidance was the major cause of female natal dispersal in this species based on the finding that females preferred to emigrate to patches providing mating opportunities, and dispersive females were more likely to mate than philopatric females. *Habrobracon hebetor* is another braconid which suffers severe inbreeding depression and a number of processes are involved in the prevention of costly sibmating in this species. Males and females typically exhibit low sexual receptivity on emergence and by the time most females are willing to mate, the majority have dispersed from the natal site (Ode *et al.*, 1995). Additionally, females avoid mating with males that developed on the same host, which may represent another strategy to avoid mating with siblings (Ode *et al.*, 1995). Protandry (or protogyny) may also result from selection against inbreeding when the sexes both disperse on emergence and come together to mate at oviposition or feeding sites, or have mating systems characterised by leks or swarms.

In gregarious species with other forms of sex determination, such as *Nasonia vitripennis*, there is currently little evidence for kin discrimination among partners or inbreeding avoidance (e.g. Shuker *et al.*, 2004). That said, there is a suggestion that female *Nasonia vitripennis* show a preference for males of a rare genotype (Grant, Snyder & Glessner, 1974; Grant *et al.*, 1980) and that such a preference may be mediated by pheromonal cues (White & Grant, 1977). However, such rare-male effects are controversial (see Partridge, 1988, for an influential critique of the rationale and experimental methodologies).

Despite the importance of interactions amongst kin in the mating systems of parasitoid wasps, the evidence that they can recognise kin and avoid mating, or even preferentially mate with them, is limited. Obligate inbreeders which suffer limited costs of sibmating (such as *Nasonia vitripennis*) would be expected to produce more daughters on mating with their brothers as they will be more related to them than to their sons. Reece *et al.* (2004) found no support for this possibility, which implies that *N. vitripennis* females cannot discriminate kin and non-kin. As males can only increase their fitness through the production of daughters, males that are somehow able to decieve females into producing more daughters (even when they are unrelated) will be at a selective advantage. More generally, the body of evidence suggests that in terms of inbreeding avoidance, mate choice may be a more passive process which relies on aspects of the mating system (such as sex-biased dispersal) to restrict an individual's potential mates (Wiley & Poston, 1996).

### (6) Post-copulatory sexual selection: sperm competition and cryptic female choice

Polyandry, which may have arisen to overcome sperm depletion, has implications for males due to sperm competition (Alonzo & Pizzari, 2013). Sperm competition occurs after copulation, when sperm of different males compete to fertilise the ova of females (Parker, 1970). In insects including parasitioids, this competition occurs within the female's reproductive tract (or body cavity if insemination is traumatic: Stutt & Siva-Jothy, 2001) and females may bias the fertilisation outcomes of sperm competition, imposing cryptic female choice on male genitalia or ejaculate characteristics (Eberhard, 1996; Simmons & Siva-Jothy, 1998; Simmons, 2001). Unsurprisingly, sperm competition and cryptic female choice are often difficult to study, not least due to the concealed nature of the processes involved (Simmons, 2001).

#### (a) Basic patterns

The most basic pattern underlying post-copulatory sexual selection is non-random ejaculate usage, often measured in terms of sperm precedence. In parasitoids, first-male sperm precedence has been established in *Trichogramma euproctidis* (Damiens & Boivin, 2005; Martel *et al.*, *b*^2008^), *Habrobracon hebetor* (Ode *et al.*, 1995) and *Nasonia vitripennis* (Holmes, 1974), while lack of sperm precedence (i.e. random sperm usage) has been found in *Anisopteromalus calandrae* Howard (Pteromalidae), Bressac, Khanh & Chevrier, 2009) and *Diachasmimorpha longicaudata* Ashmead (Braconidae), Martínez-Martínez *et al.*, 1993). In the latter case, the timing of the second mating was found to be important, with the second male siring 75% of the progeny if there was a 24 h interval between matings (Martínez-Martínez *et al.*, 1993). Similarly, in *N. vitripennis* insemination has been found to temporarily inactivate fertilisation such that eggs laid within 24 h of a mating are often unfertilised (van den Assem & Feuth-de Bruijn, 1977). If a female mated twice in quick sucession, the first male sired all the offspring, whereas if there was a gap of 3 days between matings, broods were mostly of mixed paternity (although the exact paternity success for each male was not determined; van den Assem & Feuth-de Bruijn, 1977). The second mating not only directly reduced the first male's paternity, but reduced it further by temporarily preventing fertilisation.

In addition to patterns of non-random sperm usage, we also need to know how energy is allocated into post-copulatory processes. So far, little work has been done in the parasitoids. For instance, the female reproductive tract has never been studied with post-copulatory sexual selection in mind. We do know that in *Trichogramma euproctidis* larger females have larger sperm stores (spermathecae) and that larger males have larger seminal vesicles and produce larger sperm with longer tails (Martel, Darrouzet & Boivin, 2011). However, the extent to which these patterns reflect allometry or are under (post-copulatory) sexual selection needs to clarified in the species in question. For the rest of our discussion we will follow Simmons (2001) in considering male adaptations to sperm competition to fall into two categories. Defensive adaptations (such as post-copulatory courtship and anti-aphrodisiac marking) involve avoiding sperm competition by preventing additional copulations or avoiding non-virgin females (see also Arnqvist, 1988; den Boer, Baer & Boomsma, 2010). Offensive adaptations, on the other hand, maximise male success once the competition has been joined, for instance by displacing the sperm of other males or increasing their own ejaculate size. We will consider evidence for these processes in the parasitoids in turn.

#### (b) Defensive sperm competition adaptations

Perhaps the most important defensive sperm competition is to prevent females remating. There may of course be a conflict here: a male may want to overcome the reluctance of a female in order to mate, and then make her reluctant to mate with any subsequent males. Control of remating may also be a source of considerable sexual conflict between males and females (Arnqvist & Rowe, 2005). As we have seen earlier, many female parasitoids do re-mate. Whilst this may be a result of their mating ecology, the role of males in influencing receptivity is also clear.

Male post-copulatory courtship displays are widespread in the parasitoids and many may function as a form of mate guarding. The ‘guarding-now’ hypothesis postulates that this mate guarding takes the form of physical interference with mating attempts by other males (e.g. in *Aphytis melinus* mate guarding reduces mating but does not reduce receptivity: Allen *et al.*, 1994), whereas the ‘guarding-in-absentia’ hypothesis implies reduced receptivity of females, or reduced female attractiveness to other males (King & Kuban, 2012). The post-copulatory courtship signals in a number of parasitoid wasps fits with the guarding-in-absentia hypothesis (*Nasonia vitripennis*, Barrass, 1960; van den Assem & Visser, 1976; *Lariophagus distinguendus*, Steiner *et al.*, 2008; *Spalangia endius*, King, 2010). For instance, preventing post-copulatory courtship causes the remating frequency to increase from 12.5 to 100% in *N. vitripennis* and sperm-depleted males are still able to switch off receptivity in females (van den Assem & Visser, 1976). This may explain why sperm-depleted males continue to mate, as by doing so they may reduce the number of females in the following generation (by reducing the mating success of other sperm-competent males) which will subsequently reduce the competition between their daughters and unrelated females for hosts (Damiens & Boivin, 2006).

Another way in which males can reduce the likelihood of sperm competition is by marking females with anti-aphrodisiacs, reducing their attractiveness to other males. There is currently little evidence for this in parasitoids though. In *Spalangia endius* there is evidence for a pheromonal change associated with mated females, which are less attractive to males than virgin females (King *et al.*, 2005; King, 2010). However, King & Dickenson (2008) found that it is more likely to be a change in female pheromone profile rather than pheromonal transfer from males during mating. Since this species is highly monandrous, decreased attractiveness of mated females may serve to prevent males from investing time and energy in courting unreceptive females, and may also limit female harassment, rather than being involved in preventing sperm competition (King & Bressac, 2010).

Finally, sperm competition can be avoided by preferring to mate with virgin females. Sperm competition makes virgin females more valuable as mates: mated females will already have sperm, have fewer unfertilised eggs and may be less willing to mate (King *et al.*, 2005). Not surprisingly therefore, males of some parasitoid species have demonstrated a preference for virgin females and even an aversion to mated females (see Table 2003). For instance, male *Cecidostiba semifascia* can recognise previously mated females and avoid courting them (van den Assem, 1986) and in *Spalangia endius*, males exhibit a clear preference for virgins, and an aversion to mated females (King *et al.*, 2005). Such preferences have also been suggested in both *Nasonia vitripennis* and *Trichogramma euproctidis* (Grant *et al.*, 1980; Martel *et al.*, *a*^2008^) although male *N*. *vitripennis* clearly can also attempt to mate with already mated females (e.g. the work on sperm competition and polyandry reviewed above). In terms of measuring the fitness benefits of choice, *Trichogramma euproctidis* shows first-male sperm precedence (Damiens & Boivin, 2005), and males which discriminate between mated and virgin females have greater reproductive success.

Females of many parasitoid wasp species have been found actively to release aphrodisiac pheromones, which result in sexual responses from males. In *S. endius* these pheromones also act at close range as an anti-aphrodisiac when females have already mated (and thus are no longer sexually receptive; King & Dickenson, 2008). King & Dickenson (2008) found that these pheromones were actively released by females, not passively excreted or transferred by previous mates. Dead females did not induce an aversive reaction in males, and males showed no preference for dead virgin females over dead mated females. They also found that female behaviour did not change after mating, i.e. females did not behaviourally resist male advances.

#### (c) Offensive sperm competition adaptations

In terms of offensive adaptations, there has been little work done in parasitoids. There are no recorded instances of sperm displacement in the parasitoid wasps and even in the Hymenoptera in general sperm displacement by rival males has been seldom documented [but see Shigemura & Naito, 1999 for an example in the turnip sawfly *Athalia rosae* L. (Tenthredinidae)]. Some other hymenopterans do exhibit offensive male adaptations to sperm competition however, such as attacking seminal fluid and sperm that is identified as non-self [such as in *Apis mellifera* L. (Apidae)*, Acromyrmex echinatior* Forel (Formicidae), and *Atta colombica* Guérin-Méneville (Formicidae); den Boer *et al.*, 2010)]. In *Trichogramma euproctidis* the sperm of males reared on larger hosts do have longer tails (Martel *et al.*, 2011), which in other species has been found to increase fertilisation success (e.g. nematodes, LaMunyon & Ward, 1998; bulb mites, Radwan, 1996; dung flies, Otronen, Reguera & Ward, 1997). This is clearly important in sperm competition (Fitzpatrick, Garcia-Gonzalez & Evans, 2010) but whether it serves to increase male fertilisation success in *T. euproctidis* has yet to be established empirically. Patterns of first-male sperm precedence mentioned above obviously argue against offensive sperm competition adaptations, but again much remains to be done exploring fertilisation outcomes in parasitoids.

#### (d) Strategic ejaculate allocation

Whilst males are generally considered to have far more gametes to invest in reproduction than females, sperm supplies are not unlimited. As such, strategic sperm allocation with respect to both the risk and intensity of sperm competition is predicted (Engqvist & Sauer, 2001; Parker & Pizzari, 2010; Kelly & Jennions, 2011). When sperm limitation does occur males are expected to allocate their sperm economically between females, and preferentially invest it in females which give them the greatest fitness returns (Kelly & Jennions, 2011). Perhaps some of the most compelling evidence for adaptation to sperm competition in parasitoids comes from such ejaculate modification. In *Trichogramma euproctidis*, males have been shown to transfer fewer sperm on perceiving a threat of sperm competition (i.e. when other males were present; Martel *et al.*, *b*^2008^). This species exhibits first-male precedence and so a male can optimise his reproductive success by reducing ejaculate size when the probability of obtaining fertilisations is lower. This is likely to be particularly important for prospermatogenic species, which should invest their limited ejaculates based on the probability of fertilisation success.

Males of *Dinarmus basalis* Rondani (Pteromalidae) also show evidence of facultative manipulation of ejaculate size, partially inseminating many females to enhance their fitness (Chevrier & Bressac, 2002). Strategic ejaculation by males is related to polyandry in *D. basalis* since after an initial mating the spermatheca is not full. Twenty-five percent of *D. basalis* females accepted multiple mates, but only within 2 h or so. There was no remating after 14 or 21 h and females did not replenish sperm stores when empty (Chevrier & Bressac, 2002). Females which did remate produced more daughters and became sperm depleted later in their lives. Although early remating does allow for sperm competition, males have responded to this by economising ejaculate sizes and the majority of sperm are probably utilised by the female (Chevrier & Bressac, 2002).

#### (e) Cryptic female choice

Multiple competing ejaculates faciliate cryptic female choice, where the female directly or indirectly chooses which sperm will fertilise her eggs (Thornhill & Alcock, 1983; Eberhard, 1996). However, to date the parasitoid literature regarding post-copulatory sexual selection remains somewhat male focused. Cryptic female choice might occur before, during, or after fertilisation (Eberhard, 1996). In terms of pre-fertilisation cryptic choice, females might eject the sperm of a male prior to fertilisation, discriminating against specific males. There have been no reports of sperm ejection in parasitoids and *Nasonia vitripennis* females do not appear to eject sperm even when mating with a heterospecific (Geuverink *et al.*, 2009). Females could also fail to store sperm from certain males (contributing to so-called ‘mating failure’: Rhainds, 2010). Again, although there are anecdotal accounts of mating failures in parasitoids (we see it in our own *Nasonia* stocks: R. A. Boulton & D. M. Shuker, personal observations), systematic analysis of mating failure not associated with sperm depletion remains to be conducted. Similarly, there is no good evidence yet for the idea that the female reproductive tracts represent hostile environments for sperm, which might selectively favour some sperm haplotypes over others (Wedekind, 1994; Eberhard, 1996). If anything, stored sperm probably survive quite well, as may be expected amongst generally monandrous species. For instance, sperm are stored for at least 10 days after mating in *N. vitripennis* with no adverse effects (Geuverink *et al.*, 2009) and we suspect that this is the norm. Instead, females have only really been studied in terms of whether or not they induce post-copulatory sexual selection on males (i.e. by mating multiply) and not whether they play a more active role in biasing paternity or resisting male coercion strategies.

## VII. CONCLUSIONS

(1) The parasitoid wasps are a diverse taxonomic group, with their distinctive life histories influencing mating-system evolution and patterns of sexual selection. Key aspects of their biology include structured mating populations, inbreeding, and often highly dispersed resources needed by females.
(2) The haplodiploid genetic system offers a unique perspective for studying sexual selection and sexual conflict. Parasitoid wasps are far from being unaffected by sexual selection, but this has as yet not been fully exploited by sexual selection researchers. There are a number of examples of unexplained sexual dimorphisms across the parasitic Hymenoptera, and we suspect that many more await (re)discovery. These species may provide novel tests of sexual selection theory, not least because haplodiploid genetics changes how indirect benefits accrue to choosy individuals. Haplodiploid genetics also greatly enhances genetic analysis, such that ‘genetic quality’ may be more easily measured in haplodiploids.
(3) Research has been conducted into various aspects of sexual selection in parasitoid wasps, but a number of neglected areas remain. These include pre-copulatory intra-sexual competition (in both sexes), offensive sperm competition adaptations (as well as male mate choice beyond adaptations to sperm competition, i.e. preference for virgins) and cryptic female choice. On the other hand, there has been growing interest in the role of sperm depletion in parasitoid mating systems, and its consequences for parasitoid reproductive biology (including sex allocation).
(4) The extent to which female parasitoids are polyandrous both limits certain processes of sexual selection (most obviously post-copulatory sexual selection) but also provides some of the best opportunities for using parasitoids to test more general theories of sexual selection and the evolution of mating systems. For instance, what factors favour the origin of polyandry, not just its maintenance? There is clear scope for studies utilising experimental evolution to make progress exploring why polyandry evolves from monandry.
